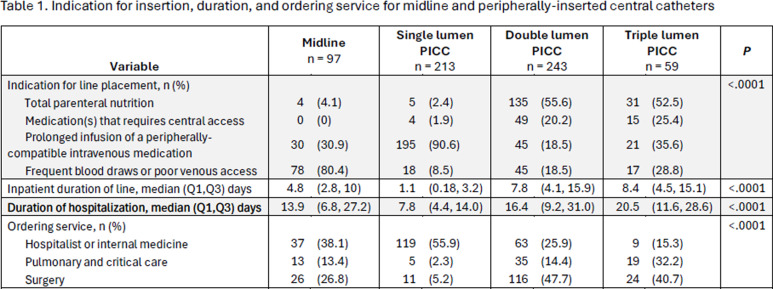# 158 Using Generative Artificial Intelligence in Hospital-onset Bacteremia and Fungemia Review

**DOI:** 10.1017/ash.2026.10559

**Published:** 2026-06-23

**Authors:** Heather Young, Rohana Bruker, Kelly Gettman, Emi Grant, Timothy Jenkins, Jeremy Nelson, Guillermo Rodriguez Nava, Ellen Sarcone, Heidi Wald, Ashlie Watters, D. Dante Yeh

**Affiliations:** 1 Denver Health Medical Center; 2 Denver Health Hospital Authority; 3 Denver Health; 4 Denver Health and Hospital Authority

## Abstract

**Background:** Midlines and peripherally-inserted central catheters (PICC) have increased in use due to their ease of insertion, low insertion risk to patients, and relatively low cost. Three common complications are central line-associated bloodstream infection (CLABSI), upper extremity deep venous thrombosis (DVT), and lumen occlusion. The literature reports PICC-associated CLABSI rates of 0.5-2.1 per 1000 catheter days, DVT rates of 1.4 to 9.5%, and lumen occlusion 5 to 16.1%. Both CLABSI and lumen occlusion are more common in multi-lumen than in single lumen (SL) PICC. Our objective is to evaluate the appropriateness of midline and PICC lumen selection, to assess for rates of complication, and to identify targets for clinical decision support intervention. **Methods:** Setting. This is a retrospective cohort study of adult patients ≥18 y.o. with a midline or PICC placed by the Vascular Access Team (VAT) between 1/1/24 and 6/30/25 at a 500-bed academic safety net and teaching hospital. Data abstraction. Indication for line placement was determined by VAT consultation order or manual chart review. Upper extremity DVT was screened by ICD10 codes and confirmed through manual chart review. CLABSI was determined through cross-reference to National Healthcare Safety Network data. Alteplase administration was used as a surrogate for lumen occlusion. Appropriateness criteria. Appropriateness of midline and PICC lumen selection was determined by adapting the Michigan Appropriateness Guide for Intravenous Catheters and Michigan Multi-Lumen Appropriateness Criteria (Figure 1). **Results:** Six hundred twelve midline (n=97, all SL) or PICCs (n=515) were placed into 524 patients in the study period (SL PICC, n=213; double lumen [DL] PICC, n=243; triple lumen [TL] PICC, n=59). The indication for line differed based on the line type (Table 1). The majority of midlines (95.9%) and SL PICCs (99.5%) were deemed appropriate; in contrast, 74.1% of DL PICC and 3.4% of TL PICC were considered appropriate. Complications of midline and PICC were rare (CLABSI: 0.19 per 1000 line days; DVT: 1.1%; lumen occlusion: 11.8%). Any complication occurred in 12.9% of cases and was more common in multi-lumen as compared to SL devices (i.e. midline or SL PICC) (19.9% vs 6.1%, P). **Conclusion:** Opportunities exist to reduce multi-lumen PICC insertion. This may decrease device-related complications. Optimizing lumen selection through clinical decision support may improve patient safety and vascular access outcomes.

**Figure:**
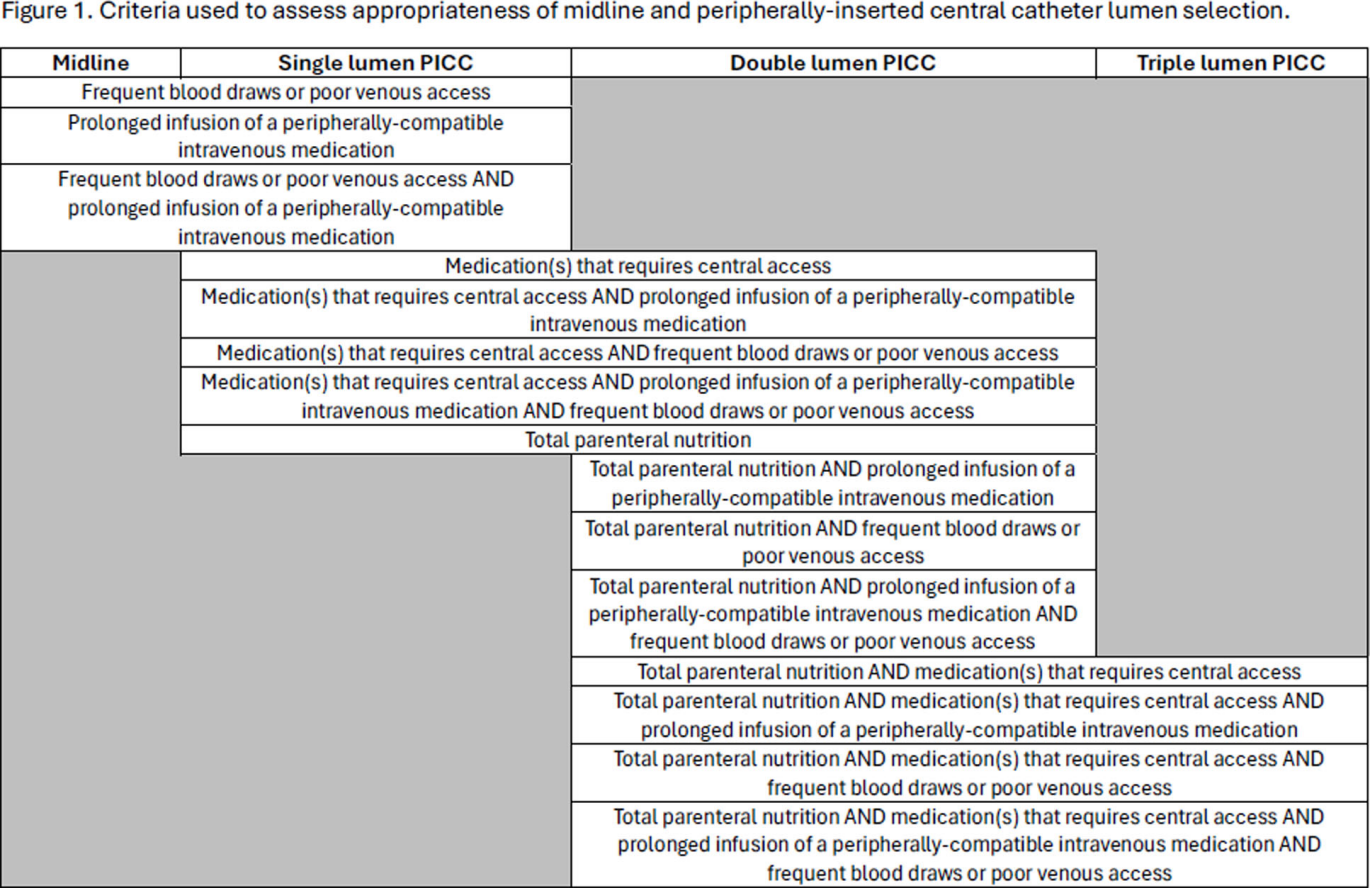

**Figure:**